# Bacterial Characteristics of Intestinal Tissues From Patients With Crohn’s Disease

**DOI:** 10.3389/fcimb.2021.711680

**Published:** 2021-11-16

**Authors:** Desen Sun, Xiaolong Ge, Shasha Tang, Yaxin Liu, Jun Sun, Yuping Zhou, Liang Luo, Zhengping Xu, Wei Zhou, Jinghao Sheng

**Affiliations:** ^1^ Department of Gastroenterology, The Affiliated Hospital of Medical School, Ningbo University, Ningbo, China; ^2^ Institute of Environmental Medicine, Zhejiang University School of Medicine, Hangzhou, China; ^3^ Department of Biochemistry and Molecular Biology and Zhejiang Key Laboratory of Pathophysiology, Medical School of Ningbo University, Ningbo, China; ^4^ Department of General Surgery, Sir Run Run Shaw Hospital, Zhejiang University School of Medicine, Hangzhou, China; ^5^ Department of Gastroenterology, Sir Run Run Shaw Hospital, Zhejiang University School of Medicine, Hangzhou, China

**Keywords:** Crohn’s disease, intestinal tissue-associated bacteria, noninflamed mucosa, inflamed mucosa, creeping fat

## Abstract

**Background and Aims:**

It is believed that intestinal bacteria play an indispensable role in promoting intestinal inflammation. However, the characteristics of these tissue-associated bacteria remain elusive. The aim of this study is to explore the bacterial loads, compositions, and structures in the noninflamed mucosa, inflamed mucosa, and creeping fat taken from patients with Crohn’s disease (CD).

**Methods:**

Noninflamed mucosa, inflamed mucosa, and creeping fat samples were obtained from 10 surgical patients suffering from CD. Total bacterial DNA was extracted in a sterile environment using aseptic techniques. The V3–V4 regions of bacterial 16S rDNA were amplified and analysed using standard microbiological methods. qPCR was used to confirm the change in abundance of specific species in additional 30 independent samples.

**Results:**

Inflamed mucosa exhibited the highest bacterial load (3.8 and 12 times more than that of non-inflamed mucosa and creeping fat) and species diversity. The relative abundance of Proteobacteria was dominant in most samples and was negatively associated with Firmicutes. Moreover, the relative abundances of *Methylobacterium* and *Leifsonia* in creeping fat significantly increased more than twice as much as other tissue types. The bacterial community structure analysis showed that the bacterial samples from the same individual clustered more closely.

**Conclusion:**

This study reveals the significant differences in bacterial load, species diversity, and composition among different intestinal tissue types of CD patients and confirms that the bacterial samples from the same individual are highly correlated. Our findings will shed light on fully revealing the characteristics of tissue-associated bacteria and their roles in CD pathogenesis.

## Introduction

Crohn’s disease (CD), one major phenotype of inflammatory bowel disease (IBD), is a chronic inflammatory disease of the gastrointestinal tract with symptoms such as chronic abdominal pain, diarrhoea, obstruction, and/or perianal lesions (Torres et al., 2017; Roda et al., 2020). According to a recent epidemiological investigation, the prevalence of CD is highest in North America and many countries in Europe. Nevertheless, since the turn of the twenty-first century, the incidence of CD has been increasing in developing countries, such as China (Ng et al., 2018; Roda et al., 2020).

The pathogenesis of CD involves a complex interaction among susceptibility genes, environmental factors, and altered gut microbiota (Torres et al., 2017; Roda et al., 2020). It has been reported that the CD microbiome displays more severe dysbiosis than that found in healthy controls and patients with ulcerative colitis (UC), another type of IBD. The most consistent bacterial changes in CD include reduced species diversity, higher gut microbiota structure instability, lower abundance of “protective” bacteria, and the higher abundance of “harmful” bacteria (Schaubeck et al., 2016; Pascal et al., 2017). In addition to the dysbiosis found in the gut lumen, it has been suggested that intestinal tissue-associated bacteria may play an important role in CD (Yu, 2018).

The translocation of commensal bacteria from the gut lumen into the mucosa layer is common in CD patients (De Cruz et al., 2015; Libertucci et al., 2018; Olaisen et al., 2021), accompanied by dysfunction and increased permeability of the intestinal barrier (Schultsz et al., 1999; Mirsepasi-Lauridsen et al., 2019). The invading bacteria can directly stimulate immune cells to produce many proinflammatory cytokines (Peyrin-Biroulet et al., 2012), which result in aggravation of intestinal inflammation (Neurath, 2014; Roda et al., 2020). Furthermore, some bacteria penetrate the intestinal wall into internal tissues, such as creeping fat (Serena and Queipo-Ortuño, 2020), a special kind of mesenteric fat wrapping around the inflamed gut in CD patients (Serena and Queipo-Ortuño, 2020). Although the formation process and accurate function of creeping fat are largely unknown, it is widely accepted that bacterial translocation could boost inflammatory responses in creeping fat (Ha et al., 2020). Thus, intestinal tissue-associated bacteria could regulate the host immune responses and play a significant role in CD initiation and perpetuation.

Although numerous data have been generated from clinical studies and animal experiments, the composition and structural characteristics of CD intestinal tissue-associated bacteria have not been completely revealed, especially in countries with a previously low incidence of IBD such as China. Therefore, we investigated the features of intestinal tissue-associated bacteria from noninflamed mucosa, inflamed mucosa, and creeping fat in Chinese CD patients.

## Materials and Methods

### Human Samples and Ethical Approval

Ten adult CD patients (≥18 years, eight males and two females) undergoing ileal resection were recruited from the Inflammatory Bowel Disease Centre, Sir Run Run Shaw Hospital affiliated with the Zhejiang University School of Medicine. CD diagnosis and sample collection were based on a standard combination of clinical, endoscopic, histological, and radiological criteria. Fresh tissue samples were frozen at −20°C within 1 h of collection, transported on dry ice to the laboratory and stored in a −80°C freezer until further analysis. All steps are carried out in a sterile environment. Ethics approval for these studies was obtained from the Ethics Committee of Sir Run Run Shaw Hospital affiliated with Zhejiang University School of Medicine (#20200622-31).

### Tissue Bacteria Enrichment and Bacterial DNA Extraction

Tissue-associated bacterial DNA was extracted from samples by using the QIAamp DNA Microbiome Kit (Qiagen, Germany) according to the manufacturer’s protocol (Heravi et al., 2020). This kit could efficiently deplete host DNA and yield enriched bacterial DNA. In brief, approximately 100 mg of intestinal tissue was homogenised, host cells were lysed, and host DNA was digested by benzonase (human DNase) while leaving the bacterial cells intact. Furthermore, microbial cells were concentrated by centrifugation, bacterial lysis buffer was used to disrupt the bacterial cells, and bacterial DNA was extracted by using a QIAamp UCP Mini Column.

### Fluorescence *In Situ* Hybridisation

The tissue samples were fixed in 4% paraformaldehyde solution overnight at 4°C, washed, and passed through 15% and 30% sucrose solutions. Samples were then embedded in optimal cutting temperature compound (OCT, Tissue-Tek) and cryo-sectioned into 5 μm longitudinal sections (Leica, Wetzlar, Germany). Slides were equilibrated in hybridisation buffer (0.9 M NaCl, 20 mM Tris-HCl, 0.01% sodium dodecyl sulfate, 10% formamide, pH 7.5) and incubated in 10 ng/μl fluorescence *in situ* hybridisation (FISH) probes (**Table 1**) for 14 h at 42°C in a humidified chamber. Slides were then incubated for 20 min in wash buffer (0.9 M NaCl, 20 mM Tris-HCl, pH 7.5) preheated to 42°C and washed gently three times. Samples were then incubated in the dark with 10 μg/ml Hoechst 33342 in PBS for 10 min at room temperature, washed three times with PBS, and mounted in Vectashield mounting medium (Vector Labs, Burlingame, CA, USA). Images were acquired on a Nikon A1 confocal microscope. The flow chart of FISH was shown in **Supplementary Figure S1**.

**Table 1:** qPCR primer pairs and FISH probes for bacteria detection used in this study.

Target bacteria	Primer/probe	Sequence (5′ to 3′)	Reference
*Universal bacteria*	341-F	CCTACGGGNGGCWGCAG	(Fadeev et al., 2021)
	805-R	GACTACHVGGGTATCTAATCC	
	EUB338, Cy3	GCTGCCTCCCGTAGGAGT	(Bai et al., 2020)
*Methylobacterium*	Methy-F	GATCGGCCCGCGTCTGATTAG	(Dourado et al., 2012)
	Methy-R	CCGTCATTATCGTCCCGGACA	
	MB, FITC	AGCGCCGTCGGGTAAGA	(Pirttilä et al., 2000)
*Leifsonia*	Lay-F	AAGGAGCATCTGGCACCC	(Sun et al., 2019)
	Lay-R	GGGAGTCACTGGGTCACC	
	Lay-F-RC, Cy3	GGGTGCCAGATGCTCCTT	This study
*Mycobacterium avium* paratuberculosis	MAP-AV1	ATGTGGTTGCTGTGTTGGATGG	(Sharp et al., 2018)
	MAP-AV2	CCGCCGCAATCAACTCCAG	
	MAP-AV1, FITC	ATGTGGTTGCTGTGTTGGATGG	(Sharp et al., 2018)
Adherent-invasive *Escherichia coli* strain LF82	LF82-PMT-F	CCATTCATGCAGCAGCTCTTT	(Galtier et al., 2017)
	LF82-PMT-R	ATCGGACAACATTAGCGGTGT	
	LF82-AF568, Cy3	GTAGACGAAGCGCACACAGC	(Sharp et al., 2018)
*Klebsiella pneumoniae*	KP16-F	GCAAGTCGAGCGGTAGCACAG	(Gomes et al., 2018)
	KP16-R	CAGTGTGGCTGGTCATCCTCTC	

### 16S rDNA Amplicon Sequencing

The V3–V4 hypervariable region of bacterial 16S rDNA was amplified using a universal sequencing primer pairs (**Table 1**). The amplicon was sequenced by Illumina MiSeq platform at Majorbio Bio-Pharm Technology Co., Ltd. (Shanghai, China). The sequence reads were analysed by Quantitative Insights into Microbial Ecology (QIIME, http://www.qiime.org) analysis pipeline as described (Caporaso et al., 2010). In brief, FASTA quality files and a mapping file indicating the barcoded sequence corresponding to each sample were used as inputs, reads were split by samples according to the barcode, taxonomical classification was performed using the RDP classifier, and an operational taxonomic unit (OTU) table was created. Closed reference OTU mapping was employed using the Ribosomal Database Project database (RDP, http://rdp.cme.msu.edu). Sequences sharing 97% nucleotide sequence identity in the V3–V4 region were binned into OTUs. Alpha-diversity and beta-diversity analyses were calculated using QIIME.

### Real-Time Quantitative PCR Analysis

Tissue bacterial DNA was extracted as mentioned above. The relative abundances of *Methylobacterium*, *Leifsonia*, *Mycobacterium avium* paratuberculosis (MAP), *Klebsiella pneumoniae*, and adherent-invasive *Escherichia coli* (AIEC) strain LF82 were then detected by qPCR with the primer pairs shown in **Table 1**. The universal bacterial abundance served as an internal reference. qPCR was performed on a Roche (Basel, Switzerland) 480 real-time PCR system.

### Statistical Analysis

All analyses were performed with GraphPad Prism 8 (GraphPad Software). Data were presented as mean ± 95% confidence interval (CI). One-way ANOVA with Tukey’s multiple comparisons test was used to assess the difference between variables. The difference between two groups was assessed using Student’s *t*-test. The correlation between two bacterial abundances was assessed by linear regression analysis. Alpha-diversity was carried out with species rarefaction analysis, Sobs index, and Shannon index. Beta-diversity was calculated by weighted UniFrac, and the significant separation of the bacterial composition was assessed by analysis of similarity test. *p*-Value less than 0.05 was considered statistically significant.

## Results

### Patient Characteristics

The 10 ileal CD patients enrolled in this study were derived from Zhejiang Province, China. All patients had no antibiotic usage 3 months prior to surgery, but the intravenous administration of surgical antibiotic prophylaxis was used before surgical incision. These patients included two females and eight males, and the ages at diagnosis ranged from 19- to 55-year olds. Other clinical characteristics, such as disease duration, disease behaviour, and medication regimens of the 10 CD patients are summarised in **Table 2**.

**Table 2:** Characteristics of the 10 patients with CD included in the study.

Patient No.	Sex	Age at diagnosis	Disease duration years	Disease behaviour	Drug
1	M	40	2	B2	5-ASA, AZA
2	F	21	9	B3	AZA
3	F	27	9	B3	AZA
4	M	24	0.5	B2	MP, 5-ASA
5	M	19	3	B2	IFX, AZA
6	M	20	2	B2	5-ASA
7	M	47	6	B2	5-ASA
8	M	55	0	B3	–
9	M	46	1.5	B3	IFX
10	M	28	4	B2	MP

M, male; F, female; B2, stricturing; B3, penetrating; 5-ASA, masalazine; AZA, azathioprine; MP, methylprednisolone; IFX, infliximab. All patients received antibiotics at induction of anaesthesia.

### Analysis of Tissue Bacterial Burden

**Figure 1:**
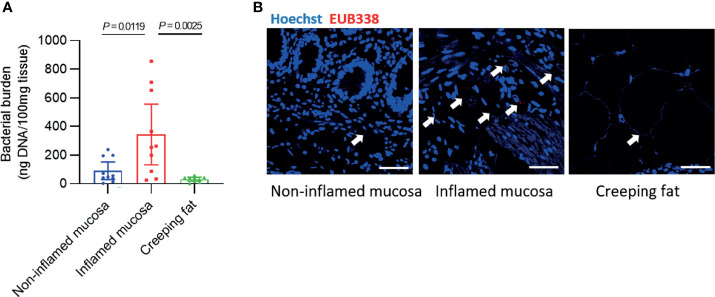
Detection of bacterial burden in different intestinal tissues. **(A)** The concentration of bacterial DNA extracted from every 100 mg of intestinal tissue. **(B)** Fluorescence *in situ* hybridisation (FISH) with an oligonucleotide probe against general bacteria (EUB338) was used to examine the number and spatial distribution of intact bacteria within noninflamed mucosa, inflamed mucosa, or creeping fat sections from CD patients: EUB338 (red) and DNA (blue). Scale bar, 100 μm. Data are presented as the mean ± 95% CI; *p*-value calculated by one-way ANOVA with Tukey’s multiple comparisons test.

We determined the total bacterial burden in each sample from the 10 CD patients. First, we used a tissue bacterial DNA extraction kit to enrich and extract intestinal tissue-associated bacterial DNA (Kiernan et al., 2019; Heravi et al., 2020). The results showed that 100 mg of noninflamed mucosal tissue contained 90.4 ± 58.5 ng of bacterial DNA, and inflamed mucosa had approximately three times more DNA (343 ± 212 ng/100 mg tissue), but only 28.8 ± 12.8 ng of bacterial DNA was found in 100 mg of creeping fat (**Figure 1A**). In addition, we used fluorescence *in situ* hybridisation (FISH) with the oligonucleotide probe EUB338, which can bind to the conserved region of 16s rRNA and recognise universal bacteria, to visualise the spatial distribution and amount of bacteria within the samples (Maire et al., 2021). The results showed that a large number of bacteria invaded into the inflamed mucosa, but only some bacteria existed in noninflamed mucosa or creeping fat (**Figure 1B**).

### Comparison of the Bacterial Composition Among Noninflamed Mucosa, Inflamed Mucosa, and Creeping Fat

**Figure 2:**
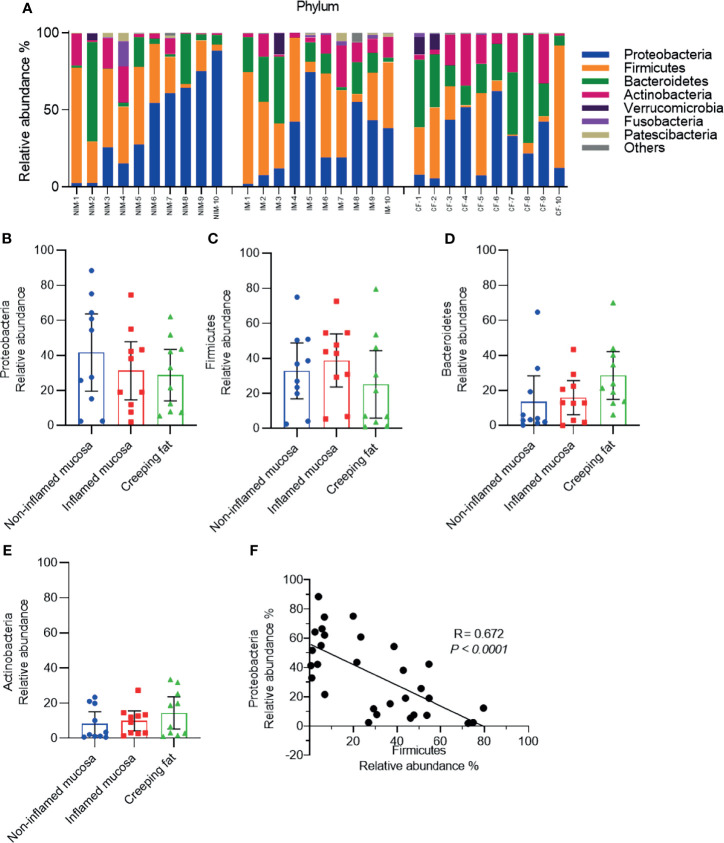
Analysis of the predominant bacterial composition at the phylum level. **(A)** Histograms of the predominant bacterial phyla in noninflamed mucosa (NIM), inflamed mucosa (IM), and creeping fat (CF) from CD patients. **(B–E)** Dynamic change in the relative abundances of Proteobacteria **(B)**, Firmicutes **(C)**, Bacteroidetes **(D)**, and Actinobacteria **(E)**. **(F)** Correlation analysis between Proteobacteria and Firmicutes. Data are presented as the mean ± 95% CI; *p*-value calculated by one-way ANOVA with Tukey’s multiple comparisons test.

To clarify the features of bacteria in noninflamed mucosa, inflamed mucosa, and creeping fat, we performed 16S rDNA high-throughput sequencing. First, taxonomic composition distribution histograms of each sample were summarised at the phylum level (**Figure 2A**). Most sequences were associated with the four dominant phyla: Proteobacteria (33.9 ± 9.2%), Firmicutes (32.3 ± 8.6%), Bacteroidetes (19.3 ± 6.8%), and Actinobacteria (10.9 ± 3.7%). However, the proportions of each phylum were significantly different from the mucosa-associated microbiota of healthy individuals, which were composed of approximately 43% Firmicutes, 33% Bacteroidetes, 10% Proteobacteria, and 5% Actinobacteria (Nishino et al., 2018). Then, we determined the dynamic characteristics of the four dominant phyla in noninflamed mucosa, inflamed mucosa, and creeping fat tissues. We noted that the relative abundance of Proteobacteria showed a decreasing trend from noninflamed mucosa to creeping fat (**Figure 2B**), the abundance of Firmicutes was highest in inflamed mucosa and lowest in creeping fat (**Figure 2C**), while that of Bacteroidetes and Actinobacteria was increased from the noninflamed mucosa to the creeping fat (**Figures 2D, E** **)**, although the between-group differences were not statistically significant. Interestingly, when we assessed the correlation between the relative abundances of the four major phyla, we found that Proteobacteria and Firmicutes displayed a significant negative correlation (*R* = 0.672, *p* < 0.0001) (**Figure 2F**), indicating that in those inflammatory microenvironments, Proteobacteria and Firmicutes compete with each other. On the other hand, no competition relationship was detected between the remaining dominant phyla (**Supplementary Figures S2A–E**). Together, these results indicate that the bacterial phyla composition is different among noninflamed mucosa, inflamed mucosa, and creeping fat tissues.

**Figure 3:**
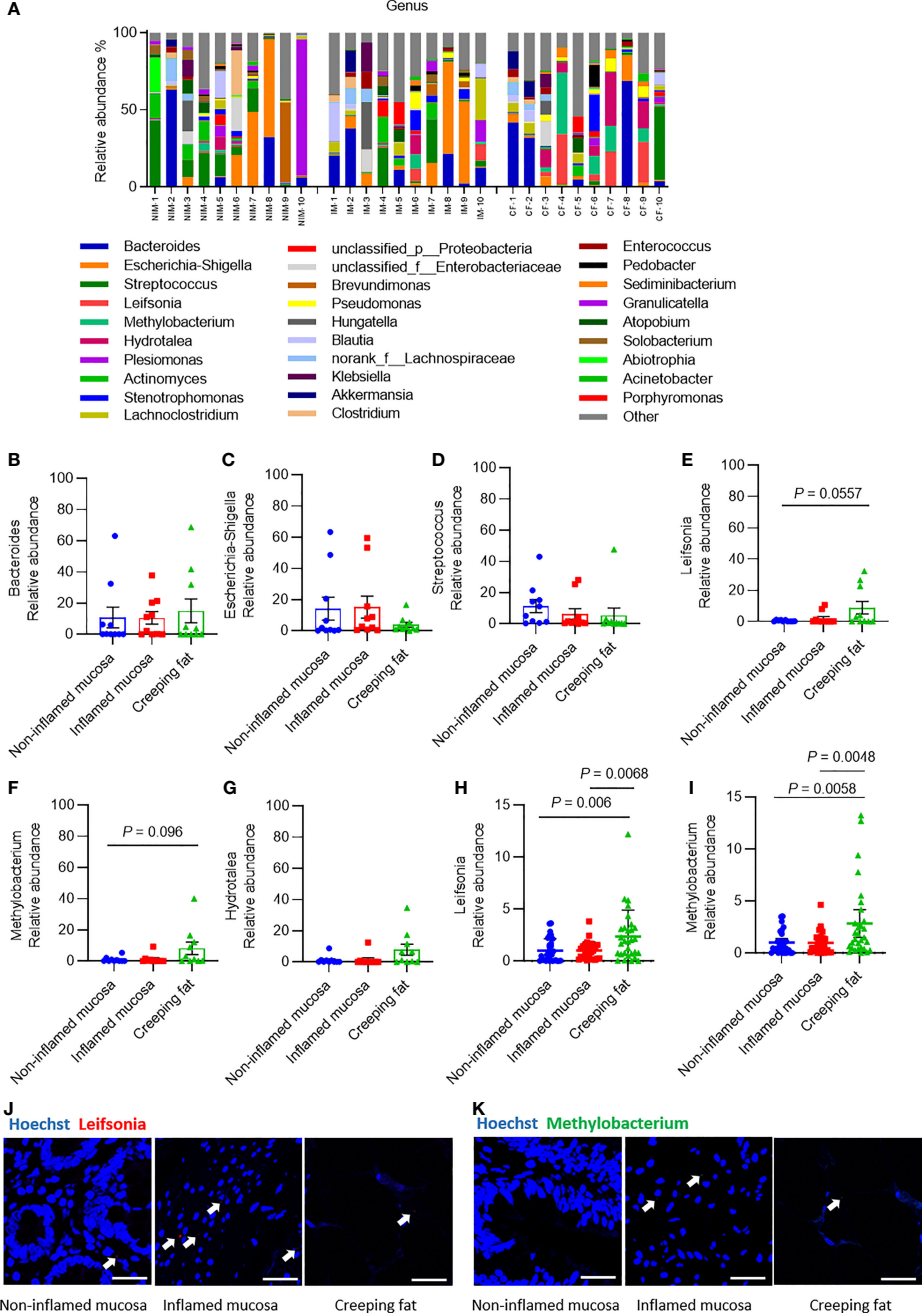
Analysis of the predominant bacterial composition at the genus level. **(A)** Histograms of the predominant bacterial genera in noninflamed mucosa (NIM), inflamed mucosa (IM), and creeping fat (CF) from CD patients. **(B–G)** Dynamic changes in the relative abundances of *Bacteroides* **(B)**, *Escherichia-Shigella* **(C)**, *Streptococcus* **(D)**, *Leifsonia* **(E)**, *Methylobacterium* **(F)**, and *Hydrotalea* **(G)**. **(H)** Relative abundance of *Leifsonia* in noninflamed mucosa, inflamed mucosa, and creeping fat detected by qPCR; *n* = 30. **(I)** Relative abundance of *Methylobacterium* in noninflamed mucosa, inflamed mucosa, and creeping fat detected by qPCR; *n* = 30. **(J, K)** FISH experiment with specific probe for *Leifsonia* and *Methylobacterium*. Scale bar, 50 μm. Data are presented as the mean ± 95% CI; *p*-value calculated by one-way ANOVA with Tukey’s multiple comparisons test.

Classification of the OTUs at the genus level resulted in the identification of many taxa, and the top 30 abundant genera are shown in histograms (**Figure 3A**). The most abundant bacterial genera were *Bacteroides*, *Escherichia-Shigella*, and *Streptococcus* (**Figures 3B–D**), consistent with previous studies (Conte et al., 2006; Kiernan et al., 2019). Although the relative abundances of all genera varied between different samples, the relative abundances of *Escherichia-Shigella* and *Streptococcus* genera showed a decreasing trend from noninflamed mucosa to creeping fat, while the relative abundances of *Leifsonia*, *Methylobacterium*, and *Hydrotalea* in creeping fat were the highest among the groups (**Figures 3E–G**). We confirmed that the relative abundances of *Leifsonia* and *Methylobacterium* were significantly increased in creeping fat by using an independent cohort of 30 CD patients’ intestinal tissues (**Figures 3H, I** **)**. The results of FISH experiment with *Leifsonia*- and *Methylobacterium*-specific probes showed that these two bacteria can invade into tissues (**Figures 3J, K** **)**.

In order to rule out the possibility of surgical contamination, we analysed the bacteria in the faecal samples collected from CD patients (*n* = 16) before surgery and healthy control people (*n* = 16). The results showed that *Methylobacterium* existed in all the CD patients’ and healthy controls’ faecal samples, while *Leifsonia* was detected in all CD and 81.25% of the healthy control (**Supplementary Figures S3A, B**). This result suggested that these bacteria already colonised in the patient’s gut before surgery was done. Furthermore, we also tried qPCR and FISH to detect whether some other well-known CD-associated bacteria, such as *Mycobacterium avium* subspecies paratuberculosis (MAP), *Klebsiella pneumoniae*, and adherent-invasive *Escherichia coli* (AIEC) strain LF82 (Naser et al., 2014; Galtier et al., 2017; Sharp et al., 2018; Keewan and Naser, 2020) existed in our CD patients’ tissue samples. The qPCR results showed that these bacteria existed in all tissue types, but the relative abundance did not differ significantly between groups (**Supplementary Figures S3C–E**); while the FISH experiment confirmed MAP and AIEC LF82 localised in the tissue samples (**Supplementary Figures S3F, G**).

### Analyses of the Diversity and Similarity of Bacterial Communities in Noninflamed Mucosa, Inflamed Mucosa, and Creeping Fat

**Figure 4:**
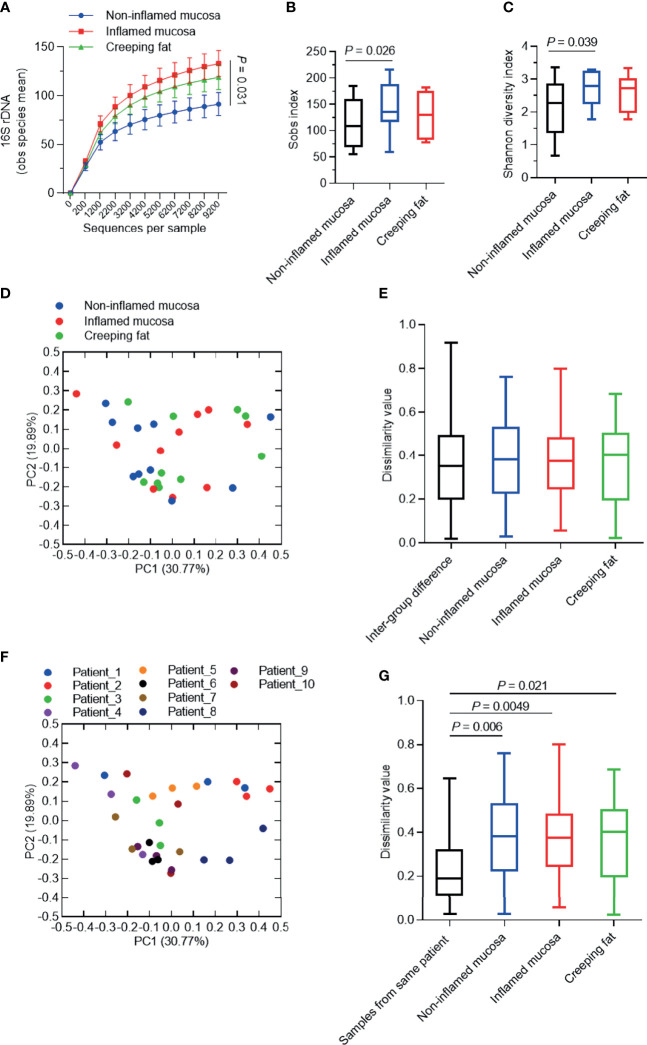
Analysis of the alpha-/beta-diversity of bacteria in different intestinal tissues from CD patients. **(A)** Rarefaction analysis of observed species mean (obs). **(B, C)** Shannon diversity index **(B)** and Sobs index **(C)** of microbial communities for noninflamed mucosa, inflamed mucosa, and creeping fat. **(D, F)** Principal coordinate analysis (PCoA) based on the weighted UniFrac difference. Samples were grouped by tissue type **(D)** or patient **(F)**. **(E, G)** Quantification of weighted UniFrac distances in **(D, F)**, respectively. The five lines of the boxplot from bottom to top represent the minimum value, first quartile, median, third quartile, and maximum value. Data are presented as the mean ± 95% CI; *p*-value calculated by paired Student’s *t*-test.

After analysing the species composition, we sought to determine whether there were characteristic changes in microbial communities in different tissue types from CD patients. Rarefaction analysis of observed species was used to compare community richness (alpha-diversity) among noninflamed mucosa, inflamed mucosa, and creeping fat. As shown in **Figure 4A**, the species richness of the inflamed mucosa group was significantly higher than that of the noninflamed mucosa group (*p* = 0.031), while the curve of the creeping fat group was just slightly lower than that of the inflamed mucosa, but with no statistically significant difference. This finding was confirmed by the Sobs index and Shannon diversity index (**Figures 4B, C** **)**.

Next, we used principal coordinate analysis (PCoA) based on the weighted UniFrac distance to investigate the community structure of microbiota in noninflamed mucosa, inflamed mucosa, and creeping fat samples. As shown in **Figure 4D**, the plot from the same sample group did not cluster together. Dissimilarity analysis indicated that the intragroup distance was not smaller than the intergroup distance (**Figure 4E**). Interestingly, when we grouped the samples by each patient, it became obvious that the samples from an individual always clustered more closely than to other samples (**Figure 4F**). Dissimilarity analysis confirmed the similarity of the samples from the same individual (**Figure 4G**). Overall, these results suggest that the vast majority of the variation observed in microbial communities occurs among individuals rather than among sample types.

## Discussion

Although it is known that bacteria exist in the intestinal tissues of CD patients, their distribution and tissue specificity remain elusive. In this study, we found that the invading bacteria are most enriched in inflamed mucosa than in two other tissue types, and bacteria can translocate into creeping fat. From the perspective of bacterial composition, our findings indicated that the proportion of Proteobacteria significantly expanded and negatively associated with Firmicutes in all tissue types; while *Methylobacterium* and *Leifsonia* were more abundant in creeping fat. We also confirmed that the bacterial community was more likely to cluster by individual patient than by tissue type. Overall, our study revealed the characteristics and differences of bacteria among different intestinal tissue types in CD patients.

Various studies have attempted to clarify whether there are differences in species diversity or bacterial load between noninflamed mucosal and inflamed mucosal tissues. However, the conclusions remain controversial: Ryan et al. (2020) and Olaisen et al. (2021) found that the microbial communities had no significant difference regardless of whether the mucosal tissue was inflamed or not; Vrakas et al. (2017) reported that inflamed mucosa had a higher bacterial burden; nevertheless, Walker et al. (2011) reported the opposite conclusion. Why were the conclusions contradictory? We thought that the sampling method difference was the main reason. Previous studies commonly use tissue samples from endoscopic biopsy, which only represent the mucosal surface, and the tissue matter collected is very limited. Here, we collected all the samples from resected bowel segments and enriched bacteria from full-thickness mucosal tissues, by which we could extract considerable bacterial DNA and obtain more accurate information about invading bacteria. Our results showed that the inflamed mucosa had a significantly higher bacterial burden and alpha-diversity than noninflamed mucosa. It was reasonable that the intestinal epithelial barrier was severely damaged by persistent inflammation, therefore, failing to prevent pathogen invasion and dissemination of commensal microbes. Notably, translocated bacteria were also detected in all creeping fat samples, although the content was low, which was different from some studies showing that only a proportion of CD mesenteric fat was positive for bacteria (Peyrin-Biroulet et al., 2012). These findings suggest that the invasion and translocation of bacteria into intestinal tissues of CD patients might be underestimated, especially in areas with inflammatory lesions.

When analysing the microbial composition, we found that the abundance of the phylum Proteobacteria was expanded abnormally in most samples, which was consistent with a previous research (Sokol et al., 2020). It was reported that an imbalanced gut microbiota often arises from a sustained increase in the abundance of the phylum Proteobacteria (Mukhopadhya et al., 2012; Shin et al., 2015), containing many conditional pathogenic species from genera such as *Escherichia*, *Citrobacter*, *Sphingomonas*, and *Brevundimonas.* Interestingly, we found that the abundance of Proteobacteria was negatively associated with Firmicutes. Utilising this competitive relationship, we might control the abundance of harmful Proteobacteria species by replenishing beneficial Firmicutes species. In fact, many experimental and clinical studies were looking for and validating probiotics that can inhibit intestinal inflammation or ameliorate IBD. Leccese et al. (2020) found that *Lactobacillus acidophilus* LA1 or *Lactobacillus paracasei* 101/37 (belonging to the Lactobacillaceae family, Firmicutes phylum) can effectively reduce adherent-invasive *E. coli* adhesion to HT29 (human colorectal adenocarcinoma cell), as well as reducing biofilm formation. Sun et al. (2021) found that *Anaerostipes* sp. and *Blautia* sp. (belonging to the Lachnospiraceae family, Firmicutes phylum) could inhibit the growth of alpha-proteobacteria in a mouse model. Evidence from clinical investigations indicated that *Lactobacillus reuteri* could reduce Proteobacteria population in the gut microbiota and improve digestive health (del Campo et al., 2014). Overall, the negative correlation between Firmicutes and Proteobacteria provides theoretical support for the development of probiotics from Firmicutes species.

At the genus level, we found that the relative abundances of *Methylobacterium* and *Leifsonia* genera were greatly enriched in creeping fat. By detecting faecal samples collected from CD patient before surgery, we confirmed that these two bacteria were truly involved in CD and excluded the possibility of contamination artefacts caused by surgery or experiment. Although this is the first time reporting that these genera could translocate into creeping fat, a previous study showed that the abundance of *Methylobacterium* was specifically increased in the submucosa compared with the mucosa in CD patients (Chiodini et al., 2015), while the abundance of *Leifsonia* was significantly increased in the mucosa-associated microbiota (Sokol et al., 2020). As creeping fat is considered an important immune regulatory organ (Peyrin-Biroulet et al., 2012; Ha et al., 2020), the effects of *Methylobacterium* and *Leifsonia* in triggering the immune responses and inflammatory process should be taken into full consideration; thus, further studies are needed to gain a mechanistic understanding of these processes.

Microbial community analysis of mucosa and creeping fat from CD patients showed that samples did not cluster by tissue type; in contrast, different tissues obtained from the same individual contained similar bacterial communities. Similarly, it has been reported previously that the mucosal and lymph node microbiota from the same patient had high correlation (O'Brien et al., 2014). Our results now confirmed that this similarity existed in noninflamed mucosa, inflamed mucosa, and creeping fat microbiome. Taking advantage of this association, we can estimate the invasive bacteria in the interior tissue by analysing the microbiomes in mucosa and then proceed with a bacterial-targeted therapeutic regimen to help control unstoppable inflammatory responses or even prevent gut origin sepsis, thereby improving patient outcomes.

In summary, our data systematically revealed the dynamic changes of bacteria in noninflamed mucosa, inflamed mucosa, and creeping fat of CD patients. Our work was essential for understanding the characteristics of intestinal tissue-related bacteria in CD patients and providing basic data for new clinical treatment strategies.

## Data Availability Statement

The datasets presented in this study can be found in online repositories. The names of the repository/repositories and accession number(s) can be found below: “https://www.ncbi.nlm.nih.gov/, PRJNA726635”.

## Ethics Statement

The studies involving human participants were reviewed and approved by the Ethics Committee of Sir Run Run Shaw Hospital affiliated with Zhejiang University School of Medicine. The patients/participants provided their written informed consent to participate in this study. Written informed consent was obtained from the individual(s) for the publication of any potentially identifiable images or data included in this article.

## Author Contributions

DS, WZ, and JHS conceived and designed the project. XG, ST, LL, and WZ recruited patients and collected tissue samples. DS, XG, ST, YL, and JS performed the experiments. DS, XG, YZ, WZ, and JHS analysed, discussed, and interpreted the data. DS and JHS wrote the original draft. ZX, WZ, and JHS wrote and reviewed the final version of the text. All authors contributed to the article and approved the submitted version.

## Funding

This study was supported by the following grants: National Natural Science Foundation of China (No. 81790631, No. 81972612, and No. 31741026), Zhejiang Provincial Natural Science Foundation of China (LQ19H030008), and Fundamental Research Funds for the Central Universities.

## Conflict of Interest

The authors declare that the research was conducted in the absence of any commercial or financial relationships that could be construed as a potential conflict of interest.

## Publisher’s Note

All claims expressed in this article are solely those of the authors and do not necessarily represent those of their affiliated organizations, or those of the publisher, the editors and the reviewers. Any product that may be evaluated in this article, or claim that may be made by its manufacturer, is not guaranteed or endorsed by the publisher.

## Abbreviations

IBD, inflammatory bowel disease; CD, Crohn’s disease; UC, ulcerative colitis; qPCR, quantitative polymerase chain reaction; FISH, Fluorescence *in situ* hybridization; OTU, operational taxonomic unit; RDP, Ribosomal Database Project; CI, confidence interval; PCoA, principal coordinates analysis; QIIME, quantitative insights into microbial ecology; MAP, *Mycobacterium avium* subspecies paratuberculosis; AIEC, adherent-invasive *Escherichia coli*; NIM, noninflamed mucosa; IM, inflamed mucosa; CF, creeping fat; M, male; F, female; 5-ASA, masalazine; AZA, azathioprine; MP, methylprednisolone; IFX, infliximab.
