# Supplementary figures and images for: Bacterial Characteristics of Intestinal Tissues From Patients With Crohn’s Disease
Source: Front Cell Infect Microbiol. 2021 Nov 16;11:711680. doi: 10.3389/fcimb.2021.711680 (PMC8635149; doi:10.3389/fcimb.2021.711680)

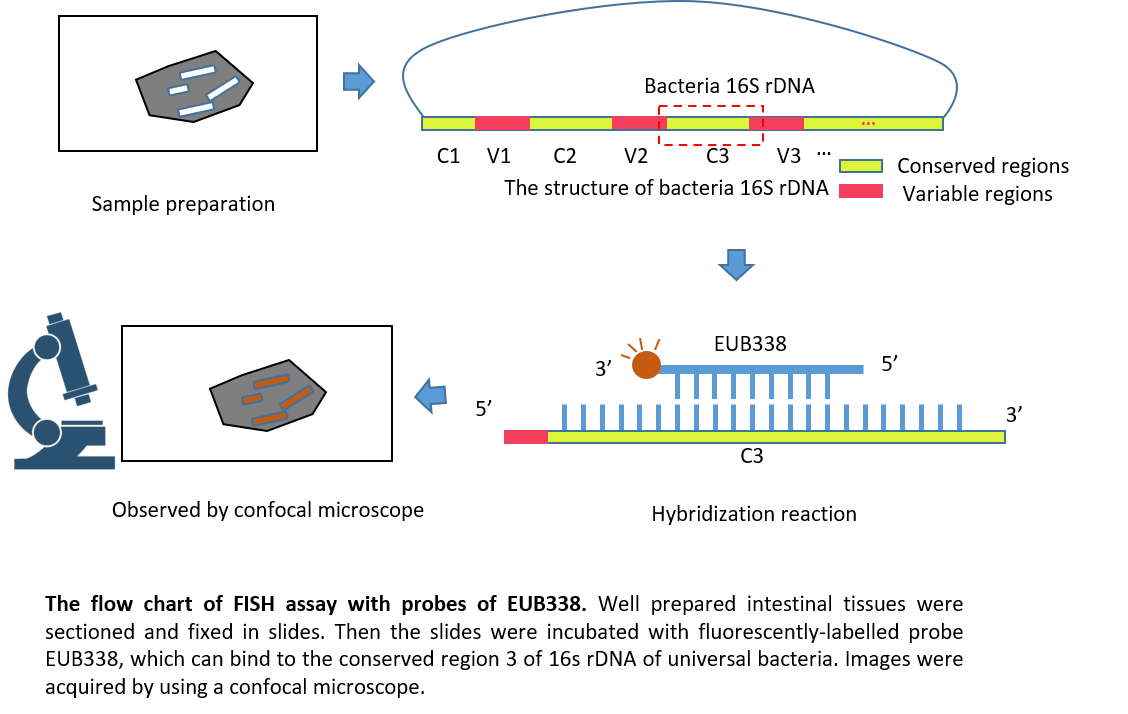

Supplement: Supplementary file 1 [file Image_1.tif]

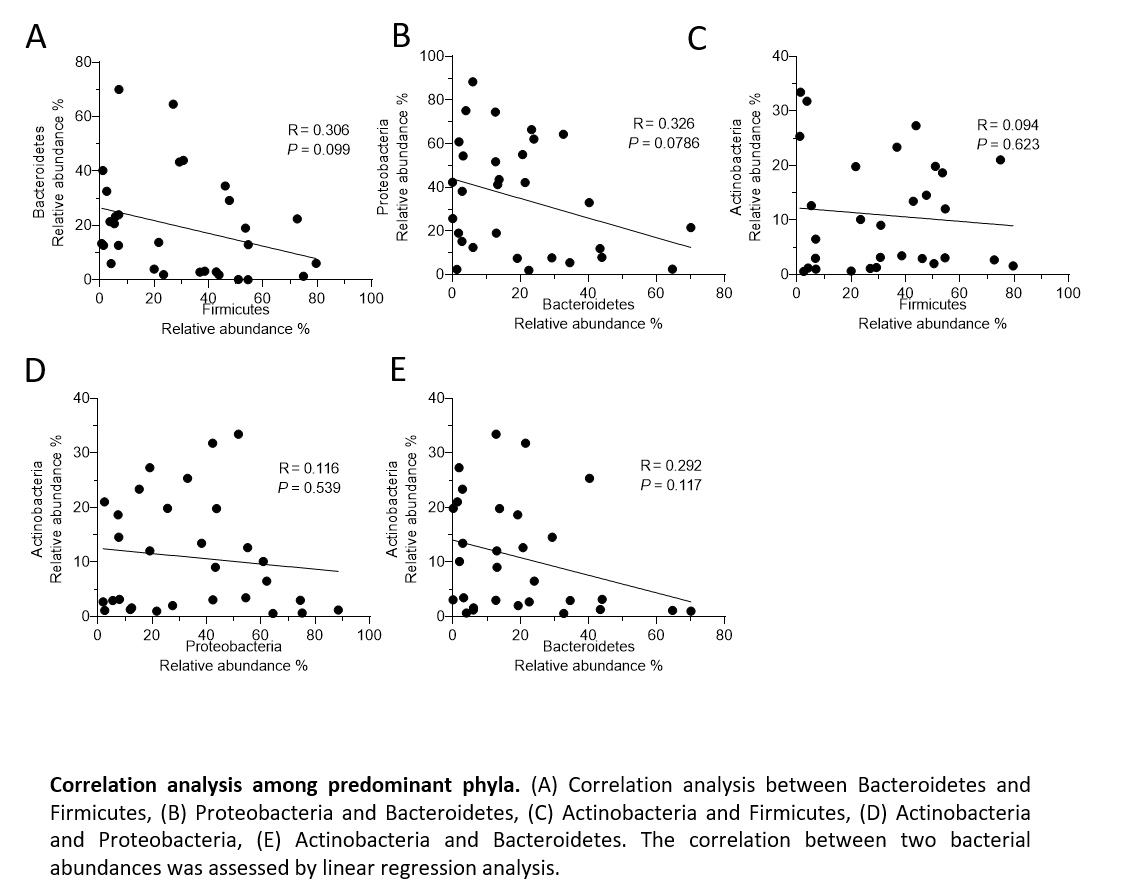

Supplement: Supplementary file 2 [file Image_2.tif]

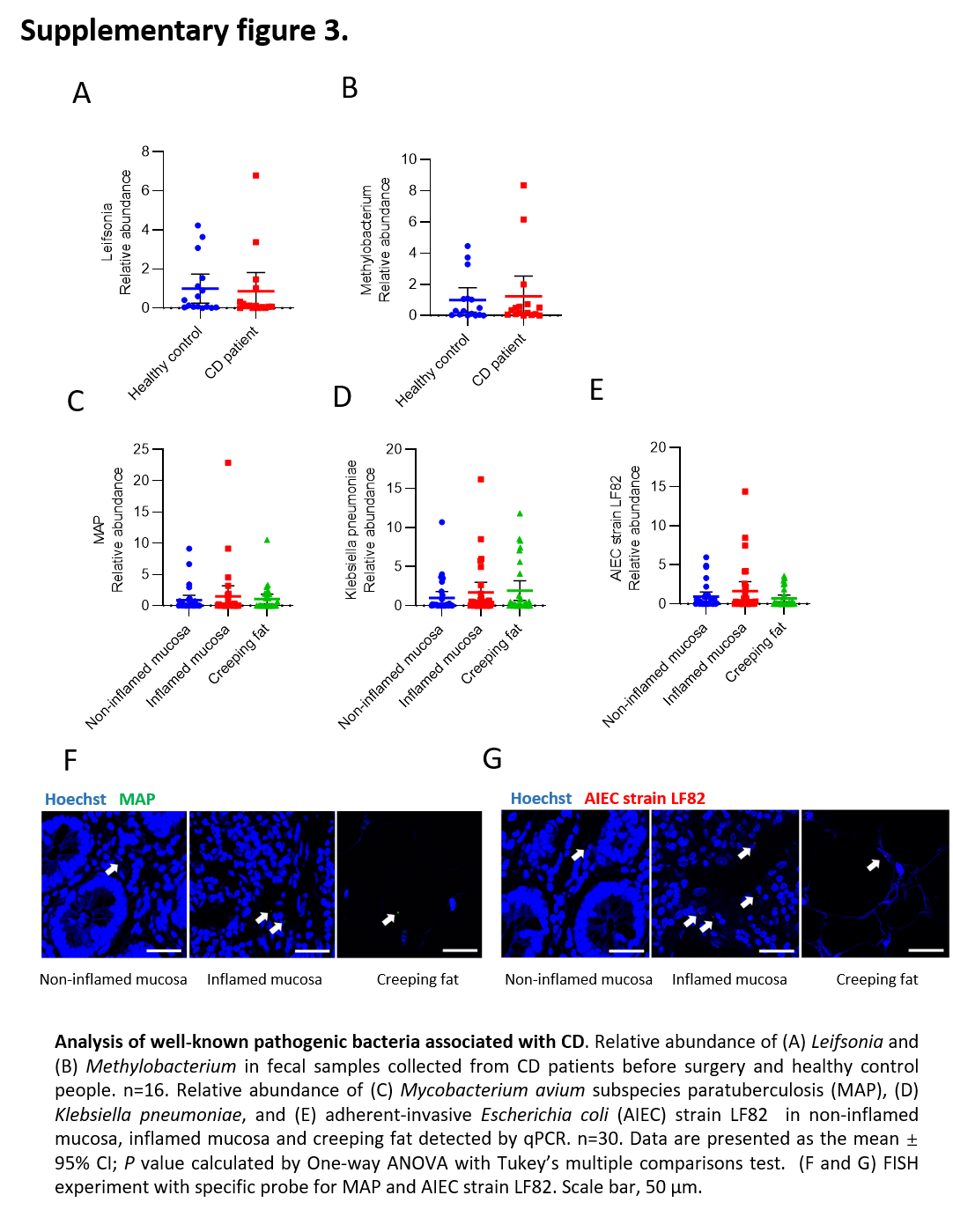

Supplement: Supplementary file 3 [file Image_3.tif]
